# Environmental Effects on Hysteresis of Transfer Characteristics in Molybdenum Disulfide Field-Effect Transistors

**DOI:** 10.1038/srep30084

**Published:** 2016-07-20

**Authors:** Yoshihiro Shimazu, Mitsuki Tashiro, Satoshi Sonobe, Masaki Takahashi

**Affiliations:** 1 Department of Physics, Yokohama National University, Yokohama 240-8501, Japan

## Abstract

Molybdenum disulfide (MoS_2_) has recently received much attention for nanoscale electronic and photonic applications. To explore the intrinsic properties and enhance the performance of MoS_2_-based field-effect transistors, thorough understanding of extrinsic effects such as environmental gas and contact resistance of the electrodes is required. Here, we report the effects of environmental gases on the transport properties of back-gated multilayered MoS_2_ field-effect transistors. Comparisons between different gases (oxygen, nitrogen, and air and nitrogen with varying relative humidities) revealed that water molecules acting as charge-trapping centers are the main cause of hysteresis in the transfer characteristics. While the hysteresis persisted even after pumping out the environmental gas for longer than 10 h at room temperature, it disappeared when the device was cooled to 240 K, suggesting a considerable increase in the time constant of the charge trapping/detrapping at these modestly low temperatures. The suppression of the hysteresis or instability in the easily attainable temperature range without surface passivation is highly advantageous for the device application of this system. The humidity dependence of the threshold voltages in the transfer curves indicates that the water molecules dominantly act as hole-trapping centers. A strong dependence of the on-state current on oxygen pressure was also observed.

Molybdenum disulfide (MoS_2_) has attracted much attention as a novel two-dimensional material for electronic and optical devices^1^,^2^,^3^,^4^. Unlike graphene^5^, MoS_2_ has a sizable energy gap, 1.2 eV for bulk and 1.8 eV for a monolayer^6^,^7^. This energy gap is highly advantageous for semiconducting device applications, including field-effect transistors (FETs)^8^,^9^,^10^,^11^,^12^,^13^,^14^,^15^,^16^,^17^,^18^. Owing to their ultrathin layered structure (0.6 nm for a monolayer), FETs with a channel made of a monolayer or few layers of MoS_2_ are immune to short-channel effects^19^. This opens the possibility of achieving a significantly high number density of FETs fabricated on a substrate, as compared to conventional Si metal-oxide-semiconductor field-effect transistors^19^.

One of the limitations of MoS_2_-based FETs is their high sensitivity to extrinsic effects such as environmental gas^9^,^15^,^17^,^18^ and contact resistance of the electrodes^20^,^21^,^22^,^23^. The environmental gas leads to instability of transport properties in the FETs. Protective passivation or encapsulation of the devices against the environment has been intensively studied^9^,^24^,^25^. On the other hand, intrinsic mobility cannot be estimated simply from the transfer characteristics acquired with two-point measurement because the contact resistance related to the Schottky barrier at the contact is typically not negligible. To explore the intrinsic properties and enhance the performance of MoS_2_-based FETs, thorough understanding of such extrinsic effects is required. In the present paper, we focus on the environmental gas effect on the transport properties of the MoS_2_-based FET. It is known that the channel surface adsorbs gases such as water vapor and oxygen, causing fluctuations in its transport properties^9^,^15^,^17^,^18^. The interaction between these molecules and the MoS_2_ surface is characterized as physisorption^26^,^27^. In particular, the hysteresis in the transfer characteristics is due to the charge trapping associated with the atoms and molecules adsorbed on the surface for MoS_2_ as well as for carbon nanotubes^28^,^29^, graphene^30^,^31^, and pentacene^32^. However, the mechanisms behind the influence of various gases have not been fully understood. In this study, we quantitatively examine the influence of various gases on the hysteresis in the transfer characteristics, such as oxygen, nitrogen, and air with varying humidities. Our results indicate that water is the main cause of the hysteresis. We observed a remarkable temperature dependence in the hysteresis, that is, the hysteresis disappeared at a modestly low temperature of ~240 K, suggesting a convenient way of suppressing the hysteresis. The dominance of hole trapping by water molecules over electron trapping is also observed.

## Results

**Figure 1:**
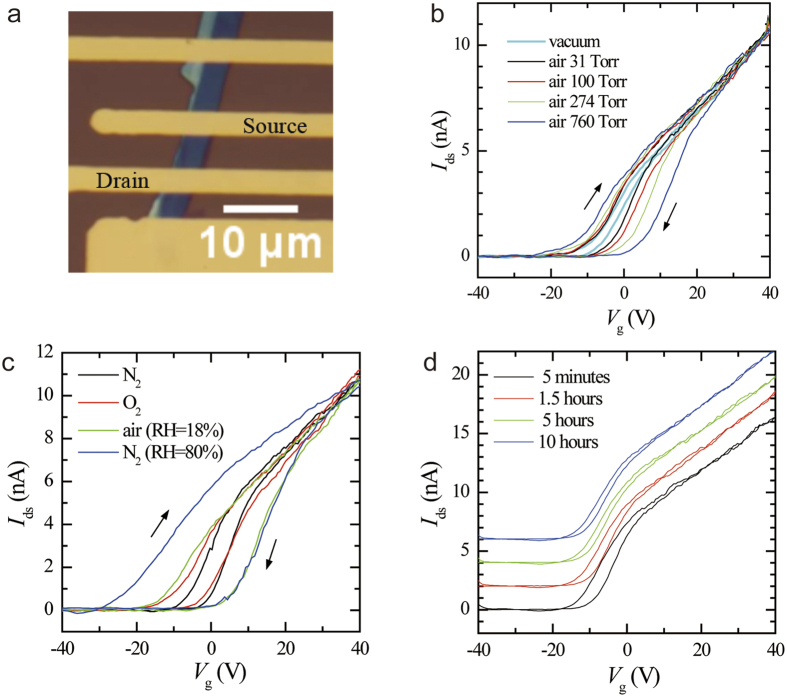
(**a**) Optical image of an FET with a multilayer MoS_2_ channel on a SiO_2_/Si substrate. The transport properties between the source and drain contacts were measured as functions of the gate voltage. (**b**) *I*_ds_–*V*_g_ curves for *V*_ds_ = 0.1 V in vacuum and air with varying values of pressure. The scan rate for the measurement of the *I*_ds_–*V*_g_ curves was approximately 1.0 V/s. The hysteresis increases with increasing air pressure. The sweep directions are indicated by the arrows. (**c**) *I*_ds_–*V*_g_ curves for *V*_ds_ = 0.1 V measured under different environmental conditions. The influence of nitrogen, oxygen, air (RH = 18%), and humid nitrogen (RH = 80%) are compared. Small temperature variation and environmental gas change the on-state current (*I*_ds_ at *V*_g_ = 40 V). Therefore, for the sake of enhancing the visibility of the hysteresis, some of the curves are vertically scaled such that the on-state currents shown in the figure nearly coincide with each other. The sweep directions are indicated by the arrows. (**d**) *I*_ds_–*V*_g_ curves for *V*_ds_ = 0.1 V measured after evacuating humid nitrogen for various periods of time using a turbo-molecular pump. The hysteresis decreases slowly; however, it persists even after pumping for 10 h. The curves are offset vertically for clarity.

**Figure 2:**
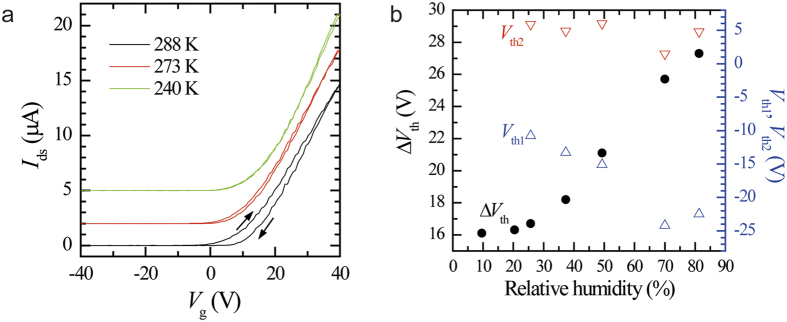
(**a**) *I*_ds_–*V*_g_ curves measured at various temperatures for a device that is different from that used for acquiring the data shown in the other figures. The thickness of the MoS_2_ flake of this device was measured to be ~5 nm. The channel length and width are *L* = 4.5 μm and *W* = 13.8 μm, respectively. In this measurement, *V*_ds_ was maintained at 1 V. The sample was cooled in helium gas for heat exchange. At 240 K, the hysteresis due to the charge trapping related to the adsorbed molecules completely disappears. The curves are offset vertically for clarity. (**b**) The threshold gate voltages *V*_th1_, *V*_th2_ (open triangles), and Δ*V*_th_ = *V*_th2_ − *V*_th1_ (filled circles) as functions of relative humidity. These data were obtained using humid nitrogen at 760 Torr as the environmental gas. The flattening of Δ*V*_th_ with decreasing humidity is explained in terms of the limited measuring range of the humidity sensor. The humidity dependence of *V*_th1_ and *V*_th2_ indicates the dominance of the hole-trapping effects of water molecules in the hysteresis of the transfer characteristics.

Figure 1a shows an optical image of the back-gated FET device with a 6-nm-thick MoS_2_ flake that was used to produce all the data except the results shown in Fig. 2a. The channel length and width of the sample are *L* = 4.3 μm and *W* = 4.1 μm, respectively. The devices with the Ti/Au contacts exhibited Ohmic *I*_ds_–*V*_ds_ behavior at room temperature, where *I*_ds_ and *V*_ds_ are the drain–source current and drain–source voltage, respectively (see Supplementary Fig. S1). Figure 1b shows the transfer characteristics (*I*_ds_–*V*_g_ curves, where *V*_g_ is the gate voltage) in vacuum and air at varying pressures. The increase in the hysteresis with increasing pressure indicates that the hysteresis is attributed to the adsorbed molecules in air that act as charge-trapping centers. The transfer curves in the different sweep directions are separately affected by the trapped charges. As the air pressure increases, the threshold voltages shift away in both the positive and negative directions, thereby increasing the hysteresis. This indicates that hole (electron) trapping is responsible for the negative (positive) shift of the transfer curve in the forward (backward) sweep, and that the time constant for charge trapping/detrapping is comparable to the measurement time of the order of 1 min. Similar results for the conductance hysteresis were obtained using fabricated FET devices with multilayer MoS_2_ channels. The transfer characteristics in dry nitrogen with varying values of pressure are compared in sFig. S2, which indicates that the main cause of the increase in the hysteresis shown in Fig. 1b is not increased nitrogen pressure.

In Fig. 1c, the transfer characteristics in different environmental gases are compared, such as dry nitrogen, dry oxygen, air (relative humidity RH = 18%), and humid nitrogen (RH = 80%). The total pressure of the gases was approximately 740 Torr for the displayed data. Table 1 summarizes the magnitudes of the hysteresis in terms of the difference in the threshold voltages Δ*V*_th_ = *V*_th2_ − *V*_th1_ in different sweep directions, where *V*_th1_ and *V*_th2_ are defined as the voltages at which the drain–source current is *I*_ds_ = 1 nA. The molecules, in decreasing order of contribution to the hysteresis, are water, oxygen, and nitrogen. It is remarkable that the influence of water (RH = 80%) on the hysteresis is more than thrice that of dry oxygen.

We note that the environmental effect on the hysteresis was reversible, that is, the hysteresis decreased again when the environmental gas was pumped out for a few minutes without annealing the devices. The small residual hysteresis in the data taken in vacuum shown in Fig. 1b is attributed to the small number of adsorbed gas molecules that remain adsorbed even in vacuum. Figure 1d shows the transfer characteristics after the humid nitrogen was evacuated using a turbo-molecular pump for various periods of time. A gradual decrease in the hysteresis is observed for a period longer than 10 h, indicating the slow desorption of water molecules in vacuum at room temperature. This is ascribed to the strong hydrogen bonds between the water molecules and sulfur surface of MoS_2_ that has a considerable polarity and hydrophilicity^9^, which is in clear contrast to the hydrophobicity of graphene and nanotubes^28^. We note that the calculated adsorption energies of water and oxygen molecules on the monolayer MoS_2_ are considerably higher than the thermal energy at room temperature^26^,^27^. The residual hysteresis in vacuum may partly be of intrinsic origin, as proposed in ref. 33.

We observe a remarkable temperature dependence of the hysteresis, as shown in Fig. 2a, using a device different from the one used for acquiring the other data. The devices had similar thicknesses but different channel lengths and widths, as specified in the figure caption. Cooling the sample below 273 K drastically reduced the hysteresis. At 240 K, the hysteresis ascribed to the charge trapping was completely suppressed. Similar temperature dependence was observed for the multiple devices we fabricated. The disappearance of the hysteresis at low temperatures is attributed to the prolonged time constant for charge trapping/detrapping. The analysis of the temperature dependence of this time constant will be a subject of future study. The suppression of the hysteresis or instability in the easily attainable temperature range without surface passivation is highly advantageous for the device application of this system.

The dependence of the magnitude of the hysteresis Δ*V*_th_ on the relative humidity is shown in Fig. 2b. A positive dependence is clearly observed up to RH = 80%. Because the measuring range of the humidity sensor used in the present study is from 20% to 85%, the saturating behavior with decreasing humidity is ascribed to the limitation of the humidity sensor. Considering Δ*V*_th_ = 2.4 V in vacuum (Table 1), Δ*V*_th_ should be approximately proportional to the relative humidity. Humidity sensors could be developed using this humidity dependence of the transfer characteristics^2^,^9^,^34^,^35^. The linearity between Δ*V*_th_ and humidity indicates the physisorption of water molecules, because the sheet density of physisorbed molecules is proportional to the gas pressure^26^.

In Fig. 2b, the threshold voltages *V*_th1_ and *V*_th2_ are also shown. With an increase in the humidity, *V*_th2_ is nearly constant; however, *V*_th1_ decreases significantly. Therefore, the increasing hysteresis is caused by the variation in *V*_th1_. Because the decrease in *V*_th1_ is caused by hole trapping in the forward sweep, this result indicates that the water molecules dominantly act as hole-trapping centers. This is also implied from the transfer curves shown in Fig. 1c. A comparison between the transfer curves in air (RH = 18%) and humid nitrogen (RH = 80%) reveals that the curve swept in the positive direction (off-to-on sweep) considerably shifts to the negative *V*_g_ direction with an increase in the RH, while the curves swept in the negative direction (on-to-off sweep) nearly coincide with each other. This observation is consistent with the results shown in Fig. 2b. We note that similar shifts in the threshold voltages, suggesting the dominance of hole trapping in the environmental effect on the MoS_2_-based FET, were indicated in several papers^9^,^15^,^18^, although this asymmetry between the electron and hole trappings was not mentioned. While it has been indicated that water and oxygen molecules act as charge acceptors in equilibrium^26^,^27^, the slow dynamics of the trapped charges on these molecules under varying gate voltage, which should explain the mechanism of the hysteresis, is still to be investigated.

**Figure 3:**
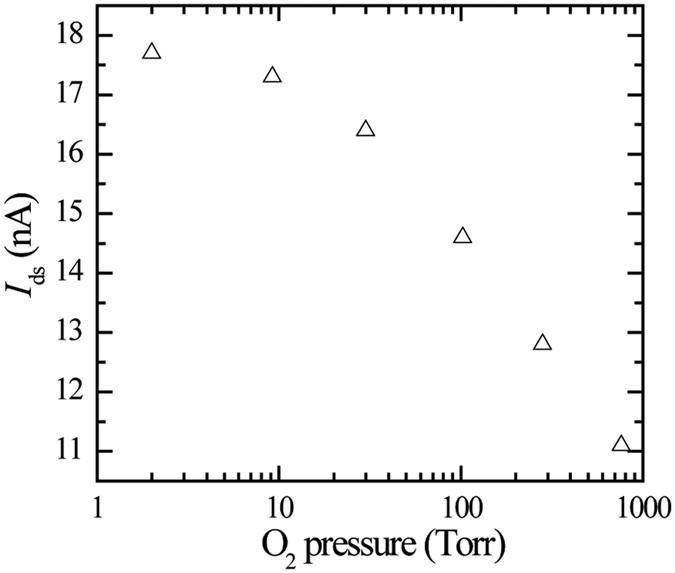
*I*_ds_ in the on-state (*V*_g_ = 40 V and *V*_ds_ = 0.1 V) measured under dry oxygen with varying values of pressure. The decrease in *I*_ds_ with increasing O_2_ pressure is explained in terms of enhanced carrier scattering due to the oxygen molecules adsorbed on the MoS_2_ channel surface.

Upon comparing the influences of various gases on the transport properties, we observed that the on-state current (*I*_ds_ at *V*_ds_ = 0.1 V and *V*_g_ = 40 V) decreased by approximately 40% with increasing oxygen pressure, as shown in Fig. 3. We note that an even stronger dependence of the on-state current on oxygen pressure was reported for a bi-layer MoS_2_ FET^18^. In contrast, the dependence of the on-state current on the RH was small (<10%) and not monotonic from RH = 10% to 80%, as shown in Supplementary Fig. S3. This small RH dependence of the on-state current may be due to the changes in temperature during measurement. The temperature dependence of conductivity is explained in terms of the Schottky barriers at the contacts and phonon scattering^17^,^18^. The small increase (14%) in the on-state current with increasing nitrogen pressure, as shown in Supplementary Fig. S4, is also assumed to be due to temperature variations. These results indicate that the adsorbed oxygen molecules on the channel surface are effective charge scatterers, while the charge scattering due to the adsorbed water molecules is not pronounced, irrespective of the fact that the charges trapped by the adsorbed water molecules are the main cause of the hysteresis in the transfer characteristics. The suppression of the scattering by the charges trapped by the adsorbed water molecules may be explained in terms of the short-range charge screening induced by the dielectric properties of the multilayer of water on the MoS_2_ surfaces, which could exist in ambient air because of hydrogen bonding among the water molecules^28^. Another possible explanation for the O_2_-pressure dependence of the on-state current is the reduction in the sheet carrier density due to the charge transfer from MoS_2_ to the oxygen molecules, which was the mechanism behind the increase in photoluminescence with increasing O_2_ pressure for the monolayer MoS_2_^26^. We assume that the reduction in the carrier density due to the charge transfer to the oxygen molecules is unlikely to be the main cause of the decreasing on-state current. This is because the MoS_2_ flakes of our devices exist in multilayers (approximately 10 layers) and the calculated values of the charge transfer to water and oxygen are not significantly different from each other (within a factor of four)^26^,^27^, while the humidity dependence of the on-state current was not observed.

## Discussion

We investigated the influence of various environmental gases, such as nitrogen, oxygen, and water, on the hysteresis of the transfer characteristics of the multilayer MoS_2_-based FETs. Water molecules are most influential on the magnitude of the hysteresis, while oxygen is less so. The dependencies on the pressure and relative humidity of the environmental gases of the hysteresis were clearly observed. While the hysteresis persisted after evacuating the device at room temperature for longer than 10 h, it was completely suppressed by cooling down to ~240 K. This suppression is attributed to the temperature dependence of the time constant for charge trapping/detrapping. The suppression of the hysteresis by modest cooling without protective passivation or encapsulation is very promising for device applications by reducing the instability in MoS_2_ devices caused by the environmental effects. The variation of the transfer curves at different levels of humidity indicates that the water molecules dominantly act as hole-trapping centers. The decrease in the on-state current with increasing oxygen pressure suggests that the adsorbed oxygen molecules become effective scattering centers for the charge carriers. These results provide a solid basis for understanding the environmental effects in the MoS_2_-based FETs. The applicability of this system to sensors for humidity and oxygen is also suggested.

## Methods

Thin MoS_2_ flakes were exfoliated from a bulk crystal (SPI Supplies) using adhesive tape^36^. The flakes on the tape were then transferred onto a gel sheet (Gel-Pak, PF-20-X4)^37^. Subsequently, the flakes on the gel sheet were deposited on a Si substrate with a 270-nm-thick SiO_2_ layer. The intermediate gel sheet step was very effective in obtaining clean flakes with very little glue on the adhesive tape^37^. After locating a suitable MoS_2_ flake using an optical microscope, the source and drain electrodes made of Ti(12 nm)/Au(75 nm) were fabricated using photolithography and electron-beam deposition. The highly n-doped Si substrate was used as a back gate. An atomic force microscope was used to measure the thicknesses of the MoS_2_ flakes, and the transfer and output characteristics of the devices were measured using a source measure unit Keithley 236 and a gate-voltage source with a homemade voltage amplifier within a ±100 V range. To examine the environmental effects, the devices were housed in a vacuum chamber with a base pressure of 10^−4^ Pa. The transport properties in different gases with various pressures were then measured. To study the influence of water molecules, water was bubbled with dry nitrogen gas. Relative humidity (RH) was monitored using a humidity sensor (TDK CHS-GSS). All the measurements were performed at room temperature except the data shown in Fig. 2a. The low-temperature data shown in Fig. 2a were taken using a cryostat with a cryogen-free refrigerator. The sample cell of the cryostat was filled with helium gas at 2000 Torr for heat exchange.

## Additional Information

**How to cite this article**: Shimazu, Y. *et al*. Environmental Effects on Hysteresis of Transfer Characteristics in Molybdenum Disulfide Field-Effect Transistors. *Sci. Rep.* **6**, 30084; doi: 10.1038/srep30084 (2016).

## Supplementary Material

Supplementary Information

## Figures and Tables

**Table 1:** Comparison of the threshold gate voltages *V*_th1_, *V*_th2_, and their difference Δ*V*_th_ = *V*_th2_ − *V*_th1_ under different environmental conditions, where *V*_th1_ and *V*_th2_ are the gate voltages at which the drain–source current was *I*_ds_ = 1 nA in the forward (positive direction) and backward (negative direction) sweep of the *V*_g_, respectively.

	*V*_th1_	*V*_th2_	Δ*V*_th_
vacuum	−8.5	−6.1	2.4
N_2_	−5.0	0.9	5.9
O_2_	−8.9	−0.5	8.4
air (RH = 18%)	−11.1	6.5	17.6
N_2_ (RH = 80%)	−22.0	6.8	28.8

Δ*V*_th_ is used as a measure of the hysteresis. The effect of hole (electron) trapping can be discussed considering the shift in *V*_th1_ (*V*_th2_) as described in the text.
