# Supplementary material for: Environmental Effects on Hysteresis of Transfer Characteristics in Molybdenum Disulfide Field-Effect Transistors
Source: Sci Rep. 2016 Jul 20;6:30084. doi: 10.1038/srep30084 (PMC4951808; doi:10.1038/srep30084)
Supplement: Supplementary Information [file srep30084-s1.pdf]

Supplementary Information

Environmental Effects on Hysteresis of Transfer Characteristics in Molybdenum Disulfide Field-Effect Transistors

Yoshihiro Shimazu, Mitsuki Tashiro, Satoshi Sonobe, and Masaki Takahashi

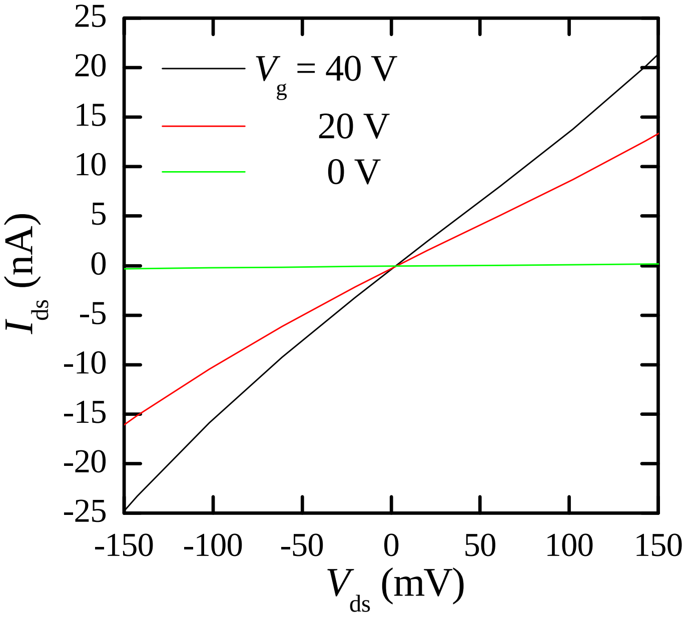

Figure S1. I_{ds}–V_{ds} curves for V_g = 0, 20 and 40 V. Ohmic characteristics are observed.

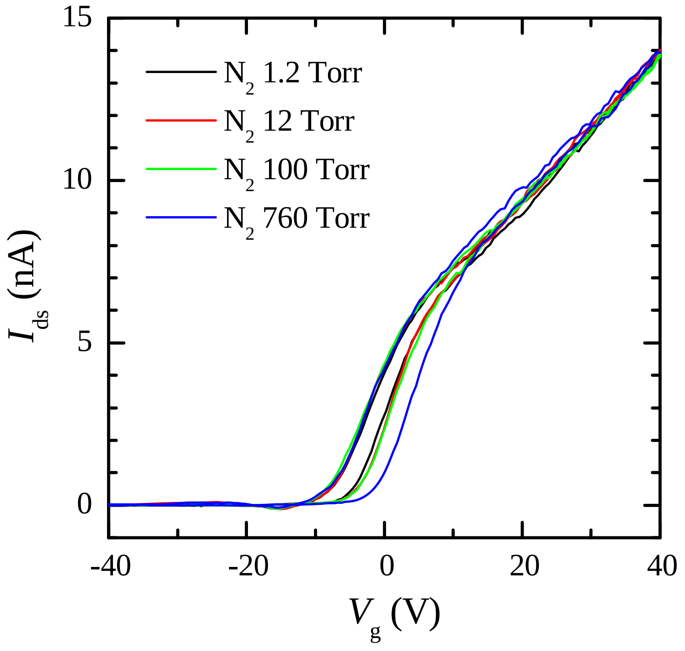

Figure S2. I_{ds}–V_g curves for V_{ds} = 0.1 V in dry nitrogen with varying values of pressure. The curves for pressures less than 100 Torr nearly coincide with each other. At 760 Torr, the increase in the hysteresis is observed.

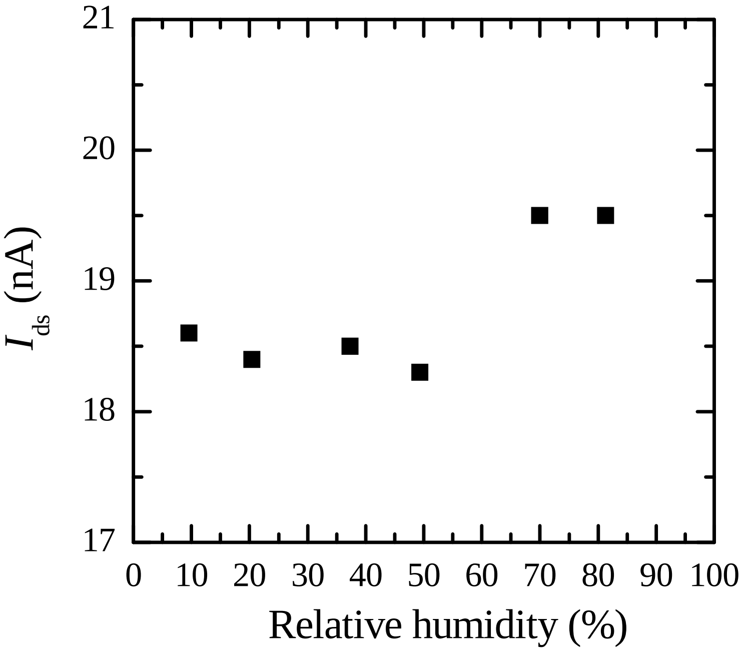

Figure S3. I_{ds} in the on-state (V_g = 40 V and V_{ds} = 0.1 V) measured under humid nitrogen condition at 760 Torr. The variation in the on-current is less than 10%.

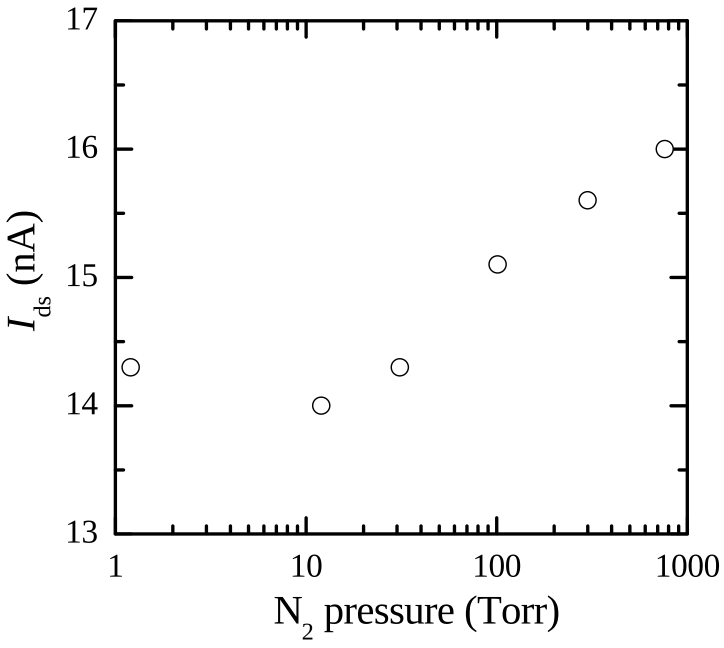

Figure S4. I_{ds} in the on-state (V_g = 40 V and V_{ds} = 0.1 V) measured under dry nitrogen condition with varying values of pressure. The on-current increases by 14% as the pressure increases from 1 to 760 Torr. The variation in the on-current shown in Figs. S3 and S4 can be attributed to the variations in temperature.
